# Identification of Serological Biomarkers for Early Diagnosis of Lung Cancer Using a Protein Array-Based Approach*[Fn FN2]

**DOI:** 10.1074/mcp.RA117.000212

**Published:** 2017-10-11

**Authors:** Jianbo Pan, Guang Song, Dunyan Chen, Yadong Li, Shuang Liu, Shaohui Hu, Christian Rosa, Daniel Eichinger, Ignacio Pino, Heng Zhu, Jiang Qian, Yi Huang

**Affiliations:** From the ‡Department of Ophthalmology, Johns Hopkins School of Medicine, Baltimore, Maryland 21205;; §Department of Pharmacology and Molecular Sciences, Johns Hopkins School of Medicine, Baltimore, Maryland 21205;; ¶Provincial Clinical College, Fujian Medical University, Fuzhou 350001, Fujian, China;; ‖Department of Clinical Laboratory, Fujian Provincial Hospital, Fuzhou 350001, Fujian, China;; **CDI Laboratories, Inc., Mayaguez, Puerto Rico 00682;; ‡‡The Sidney Kimmel Comprehensive Cancer Center, Johns Hopkins School of Medicine, Baltimore, Maryland 21205

## Abstract

Lung cancer (LC) remains the leading cause of mortality from malignant tumors worldwide. Currently, a lack of serological biomarkers for early LC diagnosis is a major roadblock for early intervention and prevention of LC. To undertake this challenge, we employed a two-phase strategy to discover and validate a biomarker panel using a protein array-based approach. In Phase I, we obtained serological autoimmune profiles of 80 LC patients and 20 healthy subjects on HuProt arrays, and identified 170 candidate proteins significantly associated with LC. In Phase II, we constructed a LC focused array with the 170 proteins, and profiled a large cohort, comprised of 352 LC patients, 93 healthy individuals, and 101 patients with lung benign lesions (LBL). The comparison of autoimmune profiles between the early stage LC and the combined group of healthy and LBL allowed us to identify and validate a biomarker panel of p53, HRas, and ETHE1 for diagnosis of early stage LC with 50% sensitivity at >90% specificity. Finally, the performance of this biomarker panel was confirmed in ELISA tests. In summary, this study represents one of the most comprehensive proteome-wide surveys with one of the largest (*i.e.* 1,101 unique samples) and most diverse (*i.e.* nine disease groups) cohorts, resulting in a biomarker panel with good performance.

Lung cancer (LC)^1^ remains the leading cause of mortality from malignant tumors worldwide (1, 2). According to the World Health Organization (WHO), among the 8.8 million cancer-related deaths in 2015, LC caused 1.69 million deaths worldwide (3). In the most populated country China, LC alone is responsible for the mortality of 42.05 per 100,000 persons (4). LC can be histologically categorized into two main classes: small-cell lung cancer (SCLC) and nonsmall-cell lung cancer (NSCLC). Approximately 79% of diagnosed LC is NSCLC, comprised of adenocarcinoma, squamous cell carcinoma and large cell carcinoma (5).

Regardless of the great advancements in targeted therapy and immunotherapy against LC in recent years, surgical resection followed by adjunctive radiation and/or chemotherapy is still the preferred method in the treatment of NSCLC patients in early stages (*e.g.* I-II LC), and when surgery is performed, there is a 70% one-year survival rate if the diagnosis is made at the earliest stage (6). Unfortunately, most LC patients are found in late stages at the time of diagnosis. For example, more than 75% of LC patients are diagnosed at more advanced stages (7). Currently, high-resolution (or low-dose) computed tomography (CT) of the chest is the only screening test shown to be efficacious at reducing mortality from early stages of lung cancer (8–10). Indeed, as reported by the National Lung Screening Trial (NLST) of randomized 53,454 high-risk, asymptomatic adults, three rounds of annual screening with low-dose CT decreased LC mortality by 20% (8). In fact, LC was only diagnosed in <2% of the participants in the low-dose CT group (11); lesions thought to be malignant often require additional invasive procedures and increased radiation exposure to confirm the diagnosis. Indeed, the cumulative risk of a false positive finding across 3 rounds of screening was 37% in the low-dose CT group at a 18% estimated overdiagnosis rate (10). Therefore, the discovery of noninvasive serological biomarkers for early stage LC diagnosis that yield high sensitivity and specificity will greatly benefit intervention and prevention of LC.

In this study, we employed a protein array-based approach to comprehensively survey autoantibodies against the human proteome for identification of novel serological biomarkers for early diagnosis in LC. Based on a screening of a large cohort of 1,101 samples, we discovered and validated a panel of three proteins, namely p53, HRas, and ETHE1, that provided 50% sensitivity and >90% specificity. ELISA tests further demonstrated the potential of this biomarker panel in future clinical diagnostic test formats.

## MATERIALS AND METHODS

### 

#### 

##### Cohort Description

All serum samples involved in this study were collected at Fujian Provincial Hospital, in Fujian Province, China, between 2014 and 2016. This cohort was comprised of 1101 serum samples collected from 162 healthy persons, 560 resident patients with LC, 153 resident patients with lung benign lesions (LBL), and 226 resident patients with other cancers. The 162 healthy persons were recruited during annual health examinations, including chest X-ray, abdominal ultrasonography, routine urinalysis, stool occult blood test, complete blood count, blood chemistries, and tumor antigen tests, such as carcinoembryonic antigen (CEA), CA199, and alphafetoprotein (AFP), to name a few. None of them showed any evidence of malignancy in the above tests. The 560 LC patients were recruited after histopathological confirmation of LC tumors. The TNM classification was used for evaluation of NSCLC staging and the VA scheme was used to classify SCLC into limited- and extensive-stages. The 153 LBL patients, including 83 pneumonia, 39 chronic obstructive pulmonary disease (COPD) and 31 pulmonary tuberculosis (TB), were recruited after accurate clinical assessment. The 226 patients with other cancers were recruited after histopathological confirmation of tumors. These patients included 34, 66, 27, 48, and 51 patients with rectal cancer (RC), liver cancer (LiC), cervical cancer (CC), esophagus cancer (EC), and gastric cancer (GC), respectively. Detailed information of each subject of this cohort is listed in supplemental Table 1. This study was approved by the Ethics Committee (*i.e.* IRB) of Fujian Provincial Hospital. The sera were prepared according to standard protocol. Five milliliters venous blood of each subject was collected into a 12.5 × 100 mm vacuum blood tube with diatomite coagulant, and centrifuged at 4000 rpm for 10 min at room temperature within 4 h after collection. Subsequently, sera were collected into 1.5 ml EP tubes and then stored at −80 °C until use.

##### HuProt Arrays and Serum Profiling Assays

HuProt arrays were provided by CDI Laboratories, Inc. Each HuProt v3.0 array is comprised of 20,240 unique human full-length proteins, covering ∼75% of the human proteome. Each serum sample was diluted 1000-fold in PBS, and profiled on HuProt arrays using a standard protocol as described previously (12–15).

##### Construction of LC Focused Arrays and Serum Profiling Assays

Candidate proteins identified in the HuProt array experiments were cherry-picked to fabricate the LC focused arrays in a 2 × 7 subarray format per slide. A 14-chamber rubber gasket (GraceBio Corp, Bend, OR) was mounted onto each slide to create individual chambers for the 14 identical subarrays on each slide. The subsequent assay process was identical to that described for HuProt array assay, with an exception that the volume of buffers or serum samples was reduced to 50 μl per subarray (12).

##### Data Analysis for Assays Performed on HuProt and LC Focused Array

First, the median values of the foreground (F*_ij_*) and background (B*_ij_*) intensity at a give protein spot (*i*,*j*) on the protein arrays (*i.e.* HuProt and focused arrays) were extracted. The signal intensity (R*_ij_*) of each protein spot was defined as F*_ij_*/B*_ij_*. Because each protein is printed in duplicate on an array, R*_ij_* was averaged for each protein as R_p_.

Z-score of each protein on protein arrays was calculated using a method similar to the one described in our previous studies (12). A stringent cutoff (Z ≥ 7) was used to determine the positives in this study. The sensitivity and specificity were calculated for each protein. For each comparison (LC *versus* negative controls), the biomarker candidates were selected with the highest discriminant ability (16), which is defined as

(Eq. 1) $$Discriminant\;ability=\frac{Sensitivity+specificity}{2}$$

For the focused arrays fabricated with the candidate biomarkers, the signal value for each protein was normalized by dividing the median value of negative controls for each sample. *p* values obtained from the *t* test were calculated and adjusted as false discovery rates (17). The optimal cutoff value for each candidate was evaluated with two criteria: 1) at least 90% specificity and 2) the highest discriminant ability.

##### ELISA Assay

To develop ELISA-based assays, p53, HRas, and ETHE1 proteins were purified from yeast as described previously (18). After 50 ng of each purified protein was coated onto individual wells of an ELISA plate, each serum sample in 1:500-fold dilution was added to carry out the standard ELISA tests (18). The immunoreactivity signals were measured by reading the A_450_.

## RESULTS

### 

#### 

##### Overall Study Design

**Fig. 1.**
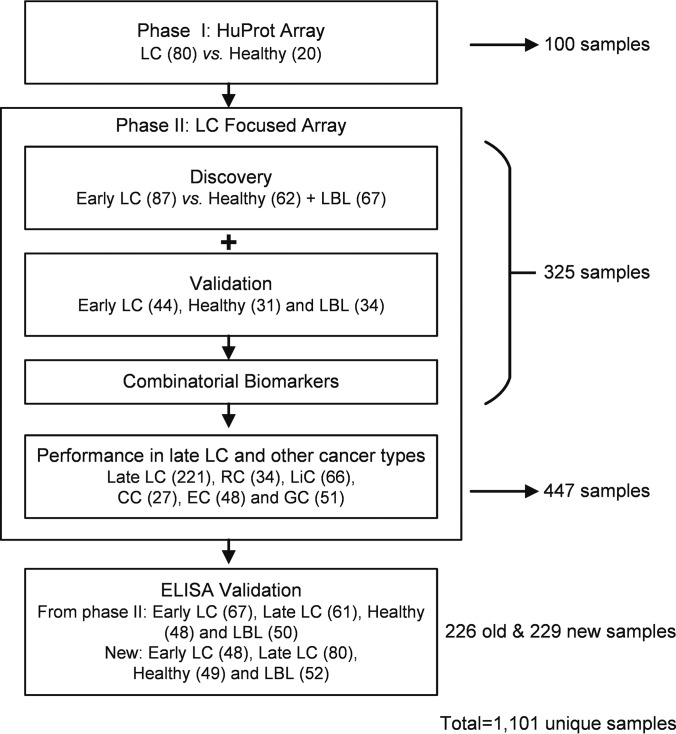
**Overall study design.**

We employed the two-phase strategy reported in our previous studies (13, 14) to identify novel biomarkers for early LC diagnosis (Fig. 1). Briefly, in Phase I, 100 serum samples collected from 80 LC patients and 20 healthy individuals, were individually profiled on HuProt arrays. After data analysis, a total of 170 candidate proteins were identified and used to construct the LC focused arrays for Phase II validation. In Phase II, we assembled a new cohort with serum samples collected from 131 patients with early stage LC and 93 healthy subjects. Because lung benign lesions (LBL) often resemble early stage LC in imaging studies, we also included 101 LBL samples as additional negative controls. We randomly split the LC samples and negative controls (healthy + LBL) in a 2:1 ratio - two thirds were used for modeling and one third for independent validation of biomarker candidates. Eight biomarkers were validated with > 13% sensitivity at > 90% specificity. Further analysis resulted in a three-protein biomarker panel with improved sensitivity, and its performance was further tested in late stage LC and other types of cancer. Finally, this panel was converted into an ELISA-based test that yielded a performance like that observed in the array-based assays.

##### Identification of Candidate Serological Biomarkers in LC Using HuProt Arrays

In Phase I, we employed HuProt arrays to profile 100 serum samples collected from 80 LC patients, including 20 SCLC, 24 adenocarcinoma, 23 squamous-cell carcinoma, and 13 large-cell carcinoma, as well as 20 healthy subjects, for candidate biomarker identification (Table I; supplemental Table S1). Statistic analyses did not show any significant differences between the LC and healthy groups in terms of age, gender or smoking history composition (Table I).

**Table I:** Characteristics of the samples in Phase I

Variable	LC (*n* = 80)			Healthy (*n* = 20)			*P*
	No.	Mean	%	No.	Mean	%	
Age (years)							0.086
Mean		60.4			56.4		
Standard deviation		8.5			11.1		
Sex							0.223
Male	66		82.5	14		70.0	
Female	14		17.5	6		30.0	
Smoking history (pack-years)							1
0	22		27.5	5		25.0	
<20	16		20.0	6		30.0	
≥20	42		52.5	9		45.0	
Type							
Small Cell Lung Cancer	20		25.0				
Large Cell Lung Cancer	13		16.3				
Adenocarcinoma	24		30.0				
Squamous Cell Carcinoma	23		28.8				

**Fig. 2.**
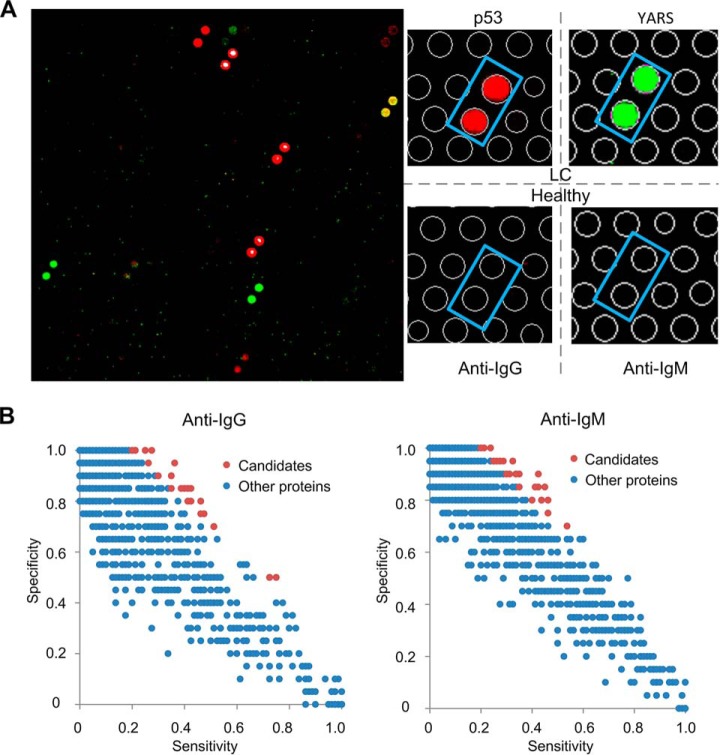
**Serum profiling assays on HuProt arrays in Phase I.** A. The left image represents serum profiling results from a portion of a HuProt array probed with Cy5- and Cy3-labeled anti-human IgG and -IgM antibodies, respectively. The right panels show two examples of positive proteins, in which p53 was only recognized by IgG antibodies of a LC patient and YARS only recognized by IgM antibodies of a LC patient. B. Scatter plot analysis of sensitivity and specificity for all the proteins on the HuProt arrays in the anti-IgG (left) and anti-IgM (right) channels. Each dot represents a protein. Red dots represent those that were selected for Phase II studies.

Each serum sample was diluted and individually incubated on the HuProt arrays, followed by a multiplexed detection of autoantigens that could be recognized by human autoantibodies of the IgG and IgM isotypes. Binding signals of both anti-IgG and -IgM channels were acquired, normalized, and quantified for each assay, based on which standard deviation (S.D.) was calculated (12). Using a stringent cut off (Z score ≥ 7), positives were determined for each serum sample. For example, p53 and YARS showed respectively strong anti-human IgG and IgM signals, mostly in LC patients, but less so in healthy subjects (Fig. 2*A*). Sensitivity and specificity values were calculated for each protein. We chose a generous criterion (*i.e.* discriminant ability ≥ 60%), resulted in identification of 170 candidate proteins, 105 and 77 of which were chosen from the anti-IgG and -IgM profiles, respectively (Fig. 2*B*; supplemental Table S2). Functional enrichment analysis identified many cancer-relevant terms, such as regulation of apoptosis and small GTPase mediated signal transduction, as well as signaling pathways relevant to cancers such as colorectal cancer, pancreatic cancer, and thyroid cancer (FDR < 0.5) (19, 20) (supplemental Table S3).

##### Identification and Validation of Biomarkers for Early Stage LC Diagnosis with LC Focused Arrays

In Phase II, we fabricated a LC focused array with the 170 candidate biomarker proteins to enable validation with a much larger cohort. We assembled a new LC cohort with serum samples collected from 131 patients with early stage LC, including 30 limited stage SCLC, 55 stage I/II adenocarcinoma, and 46 stage I/II squamous-cell carcinoma. Negative controls included 93 healthy subjects and 101 serum samples from 55 pneumonia, 26 COPD, and 20 pulmonary TB patients. Statistic analysis did not find any significant differences in age, gender or smoking history between the LC groups and negative controls (Table II; supplemental Table S1). To enable modeling and validation for biomarker identification, we randomly split each LC subgroup and negative controls in a 2:1 ratio - two thirds were used for modeling and one third for subsequent independent validation of biomarker candidates.

**Table II:** Characteristics of the samples in Phase II

Variable	Early LC (*n* = 131)			Healthy (*n* = 93)			LBL (*n* = 101)			*P*
	No.	Mean	%	No.	Mean	%	No.	Mean	%	
Age (years)										0.165
Mean		61.2			58.3			61.1		
Standard deviation		10.0			8.4			8.8		
Sex										0.102
Male	101		77.1	64		68.8	69		68.3	
Female	30		22.9	29		31.2	32		31.7	
Smoking history (pack-years)										0.108
0	24		18.3	23		24.7	28		27.7	
<20	29		22.1	24		25.8	21		20.8	
≥20	78		59.6	46		49.5	52		51.5	
Type										
Small Cell Lung Cancer	30		22.9							
Adenocarcinoma	55		42.0							
Squamous Cell Carcinoma	46		35.1							

**Fig. 3.**
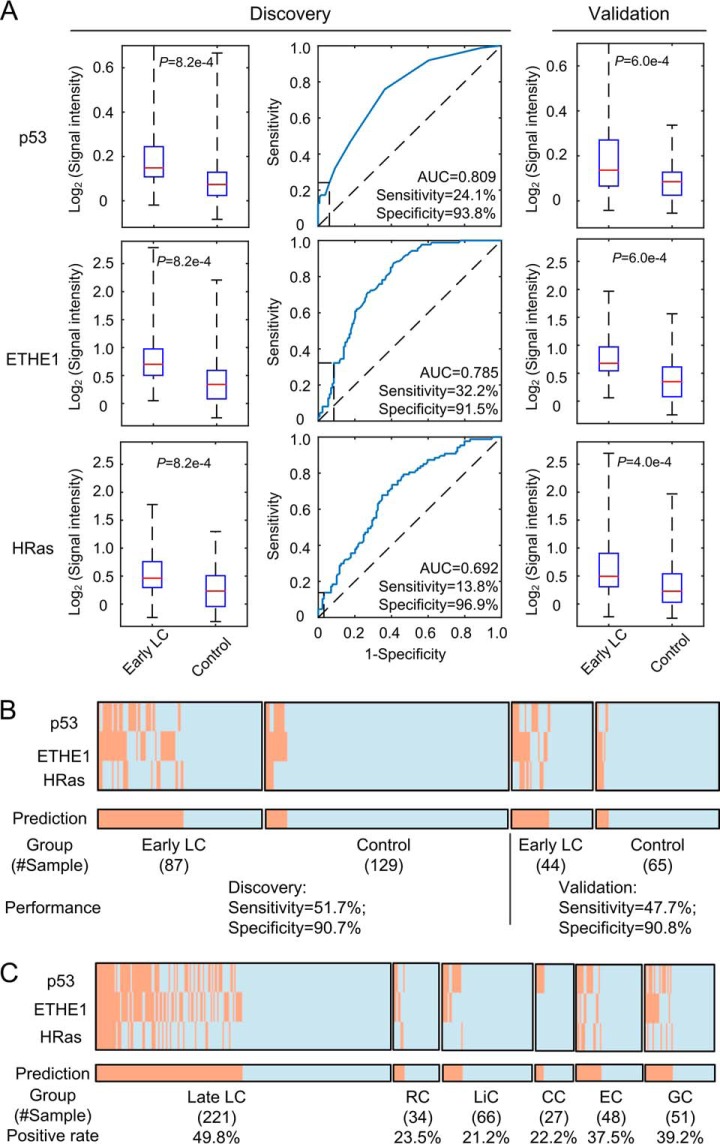
**Biomarker discovery and validation in Phase II studies.** *A*, Examples of three proteins discovered and validated in Phase II. Left panels (Discovery): Box plots show that the signal intensities of the three proteins are significantly higher in the early LC group than the control group. The corresponding ROC curves are shown to the right of the box plots. Values of sensitivity and specificity obtained at the optimal cut off value for each protein are also shown. Right panels (Validation): Box plot analysis confirmed that the signal intensities of all three proteins are significantly higher in the early LC group than the control group. *B*, Performance of the top biomarker panel, comprised of p53, ETHE1, and HRas, in the discovery and validation stages. Orange and light blue lines represent samples scored as positives and negatives, respectively. A sample was predicted as LC positive when any one of the three proteins in this panel scored positive. Values of sensitivity and specificity in both discovery and validation stages are listed below. *C*, Performance of this biomarker panel in late LC and other types of cancer. Positive rates, defined as percentage of samples scored as positives in each disease category, are shown at the bottom.

Each serum sample was profiled individually on the LC focused arrays using a similar protocol as described above. Again, both anti-IgG and -IgM profiles were obtained simultaneously. In the modeling stage, we compared the serum profiles between the LC and negative controls to identify biomarkers using stringent criteria—FDR < 0.001 and ≥ 1.20 fold-change of average signal intensity between the two groups. The analysis of IgG identified eight proteins, namely p53, ETHE1, CTAG1A, C1QTNF1, TEX264, CLDN2, NSG1, and HRas (Table III). However, the same analysis did not reveal any significant biomarkers using the anti-IgM signals. The IgG signal distributions of p53, ETHE1 and HRas in the LC and negative controls are shown as examples in Fig. 3*A*. Areas under the receiver operating characteristic (ROC) curves (AUCs) were calculated to assess the performance of each candidate biomarker. The AUC values of the eight proteins ranged from 0.68 to 0.81 (Table III). We next calculated the maximum discriminant ability values for each protein with a requirement of a minimum specificity of 90% (see Methods). This approach allowed us to determine the optimal cutoff values of signal intensity for each protein with the corresponding sensitivity and specificity values (Table III).

**Table III:** Performance of eight biomarkers in discovery and validation stages of Phase II

Protein	Discovery				Validation	
	AUC	Cut off	Sensitivity	Specificity	Sensitivity	Specificity
p53	0.809	1.209	24.1%	93.8%	22.7%	96.9%
ETHE1	0.785	1.861	32.2%	91.5%	29.5%	93.8%
CTAG1A	0.784	1.200	17.2%	96.1%	18.2%	93.8%
C1QTNF1	0.763	1.577	26.4%	90.7%	22.7%	93.8%
TEX264	0.759	2.088	23.0%	92.2%	20.5%	93.8%
CLDN2	0.744	1.820	26.4%	90.7%	22.7%	95.4%
NSG1	0.740	1.735	27.6%	91.5%	29.5%	92.3%
HRas	0.692	1.980	13.8%	96.9%	18.2%	93.8%

To validate these potential LC biomarkers, we compared the signal intensity of each protein between the LC and negative controls in the validation cohort. As visualized in the box plot analysis, all of them showed significantly higher signal intensities in the LC than the negative controls (supplemental Fig. S1). Three proteins, p53, ETHE1, and HRas, are shown as examples in Fig. 3*A*. We next applied the optimal cut-off values obtained in the modeling stage to determine the sensitivity and specificity for each protein in the validation cohort. All of the eight proteins yielded similar or better sensitivity and specificity values in the validation cohort (Fig. 3*B*; supplemental Fig. S1), confirming that the identified biomarkers have robust classification power for early stage LC diagnosis.

##### Identification of Combinatorial Biomarker Panels with Improved Performance for Early Stage LC Diagnosis

We noticed that the sensitivity values of each biomarker ranged from 13.8% to 32.2%. Therefore, we attempted to identify combinatorial biomarker panels with better performance. We exhaustively evaluated the performance for all possible combinations between two and eight proteins (=253 combinations). First, we employed a binary scoring system to convert the actual signal intensity of each protein to either 1 or 0, such that 1 represented signal intensity greater than the optimal cutoff value, and 0 otherwise. Next, we evaluated the performance of every possible combination in the discovery cohort. For a given combination of n proteins, the sum of the binary scores of the *n* proteins was assigned to each serum sample as a summary score. If the summary score of a sample was greater than k (1 ≤ *k* ≤ *n*), the sample was called positive. The sensitivity and specificity at the best discriminant ability value were recorded for each combination. Finally, we identified the combination and its *k* value with the best discriminant ability by requiring a minimum specificity of 90%.

As a result, the best combination, comprised of p53, ETHE1, and HRas, achieved 50.7% sensitivity at 90.7% specificity with a k value of 1. In other words, a serum sample would be scored positive when at least one (*i.e. k* = 1) of the three proteins showed signal intensity greater than the corresponding optimal cutoff value. When this panel was applied to the validation cohort, we obtained similar values of sensitivity and specificity (Fig. 3*B*), demonstrating the robustness of this panel in diagnosis of early LC. Moreover, after combing the results of the discovery and validation stages, the overall sensitivity for diagnosis of SCLC of limited stage and stage I/II adenocarcinoma, squamous cell carcinoma is 53.3%, 45.5% and 54.3%, respectively. When only high-risk smokers (*i.e.* ≥ 20-pack year & age > 55 years) were compared between early LC and negative controls, the performance of this biomarker panel remained almost the same at 50.0% sensitivity and 84.8% specificity.

##### Performance of the Biomarker Panel in Late Stage LC and Other Types of Cancer

To evaluate potential value of this biomarker panel in late stage LC diagnosis, we recruited a new LC cohort of 221 serum samples, collected from 43 patients with extensive stage SCLC, 99 patients with stage III/IV adenocarcinoma, and 79 patients with stage III/IV squamous-cell carcinoma, and profiled them on the LC focused arrays. By applying this biomarker panel to analyze the obtained data set, we observed a sensitivity of 49.8%, suggesting that this biomarker panel was also useful for late stage LC diagnosis.

It is known that many of the same tumor antigens can be found in patients with a wide variety of cancers, diminishing their value for accurate diagnosis of a cancer type. To evaluate the performance of this biomarker panel in other types of cancer, we profiled a cohort of 226 serum samples, collected from 34 rectal cancer (RC), 66 liver cancer (LiC), 27 cervical cancer (CC), 48 esophagus cancer (EC), and 51 gastric cancer (GC) patients, on the LC focused arrays. Interestingly, this biomarker panel could only detect 23.5%, 21.2%, 22.2%, 37.5%, and 39.2% of RC, LiC, CC, EC, and GC, respectively (Fig. 3*C*). This comparison indicated that this biomarker panel is clearly more sensitive in detecting LC.

##### ELISA Validation of the Biomarker Panel

**Fig. 4.**
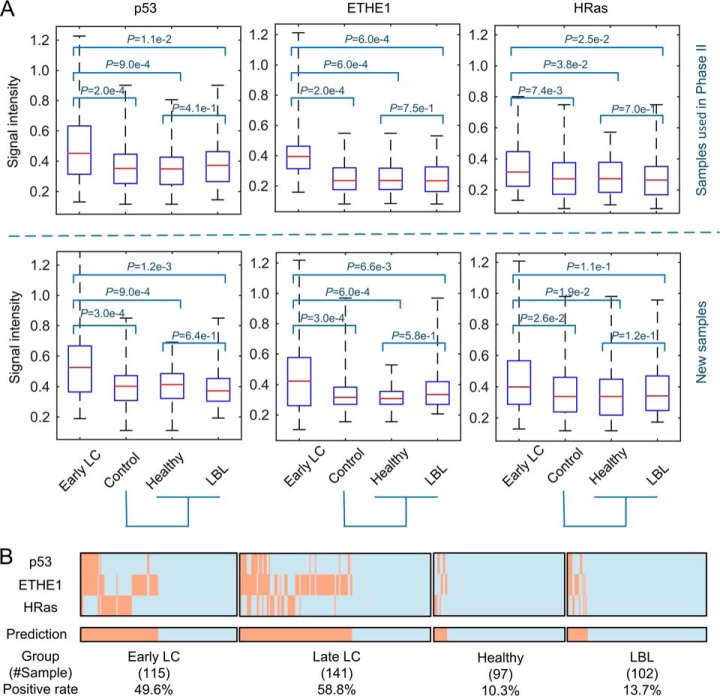
**ELISA validation.** *A*, Box plot analysis of ELISA data obtained with samples used in Phase II study (upper panel) and newly recruited samples (lower panel). *p* values were calculated between the early LC and control groups (*i.e.* healthy and LBL). *B*, Performance of this biomarker panel in early LC, late LC, healthy, and LBL groups. Positive rates of each category are shown at the bottom.

To transform the array-validated biomarker panel into a more clinically friendly platform, we developed an enzyme-linked immunosorbent assay (ELISA) for the three proteins. Two cohorts were assembled: one contained 226 samples randomly selected from those used in Phase II and 229 newly collected samples (see Fig. 1; supplemental Table S1). As expected, analysis of the ELISA data obtained with the samples used in the array-based assays demonstrated that all three proteins showed significantly higher signals in both early and late LC groups as compared with those in healthy and LBL groups. To ensure more rigorous tests, the 229 newly collected samples were tested in a single-blind fashion. A similar result was obtained (Fig. 4*A*).

We next evaluated the performance of this biomarker panel with the combined ELISA data sets. The ELISA data were converted to a binary scoring system by using a cut off value of 2-S.D. above the mean of the signal intensity of the combined healthy group, following the standard ELISA protocol. Using the same criteria as described above, 49.6% and 58.8% of samples in the early and late stages of LC, respectively, were scored as positives (Fig. 4*B*). In contrast, only 10.3% and 13.7% of healthy and LBL samples were respectively scored as false positives. Therefore, this biomarker panel showed 49.6% sensitivity at 87.9% specificity for early LC diagnosis in the ELISA tests. Moreover, the overall sensitivity obtained in the ELISA tests for diagnosis of SCLC of limited stage and stage I/II adenocarcinoma, squamous cell carcinoma is 55.9%, 44.4% and 48.9%, respectively.

## DISCUSSION

Our study design possessed and displayed several strengths. First, we employed the most comprehensive human proteome (HuProt) arrays, with >75% coverage of the human proteome to improve the likelihood of finding potential biomarkers. Second, we recruited 560 LC patients with SCLC and NSCLC who presented with all three forms at different disease stages, aiming at finding robust LC biomarkers. Third, we combined the LBL samples with healthy subjects as negative control groups to enable better discrimination of malignant from benign lesions. Finally, ELISA was used as an independent platform to evaluate the performance of the newly discovered biomarker panel. A limitation of this study is that only Chinese serum samples were employed, raising a possibility, though remote, that there could exist some ethnic bias. Therefore, further validation studies with serum samples collected from other ethnic groups are necessary to confirm the performance of this biomarker panel.

This design allowed us to rapidly discover and validate eight proteins, namely p53, ETHE1, CTAG1A, C1QTNF1, TEX264, CLDN2, NSG1, and HRas, as biomarkers for LC early diagnosis. Many of them are highly relevant in tumorigenesis. For example, p53 is a very well studied tumor suppressor involved in a plethora of cellular functions, such as inducing cell cycle arrest, apoptosis, senescence, DNA repair, or changes in metabolism (21, 22). Many mutations in p53 are found in various types of tumors, including LC (23, 24). HRas is a member of the Ras oncogene family. Somatic mutations in *HRAS* have been found to be associated with bladder cancer, thyroid carcinoma, salivary duct carcinoma, epithelial-myoepithelial carcinoma, and kidney cancers (25, 26). ETHE1 is a member of the metallo beta-lactamase family that catalyzes the oxidation of a persulfide substrate to sulfite (27). This protein has not been reported as a biomarker for any diseases to the best of our knowledge. Interestingly, ETHE1 has been shown to suppress *TP53* expression via formation of a protein complex with HDAC1 and p53 (28). This observation might provide novel insights into the etiology of LC development. CTAG1A is a known tumor cell antigen found in various types of cancers (29). Furthermore, seven of the eight biomarkers (except CTAG1A) showed positive immunohistochemistry staining in LC tissue sections (30). In addition, to our disappointment, none of the candidate biomarkers identified in Phase I could be validated in the anti-IgM profiles in Phase II. One possible explanation for this inconsistence might be the fact that the more generous criteria (*e.g.* lower specificity and sensitivity required) were used for selecting candidate biomarkers in Phase I because we intended to be more inclusive for not missing any potential candidates. The fact that none of the anti-IgM candidates could be validated in Phase II emphasizes the importance of implementing an independent validation step in biomarker discovery.

The biomarker panel identified in this study outperformed previously reported LC biomarkers. For example, the sensitivity of detecting circulating tumor antigens, such as CA125, CA199, neuron specific enolase (NSE), carcinoembryonic antigen (CEA), and cytokeratin 19 fragment (CYFRA 21–1) is only 5.0%, 4.9%, 19.7%, 17.2%, and 26.5%, respectively, in patients with stage I NSCLC (31). In addition, the fact that some of these tumor antigens, such as CYFRA 21–1, are found elevated in serum samples of patients with radiation pneumonitis has limited their use in distinguishing LC from pneumonitis (32, 33). Finally, the concentration of many circulating antigens tends to be very low because only a fraction of these proteins is distributed to the plasma from a few cancer cells in the preclinical stage, making it extremely challenging to detect them (34, 35).

The adaptive immune system is able to effectively amplify and memorize immune responses to tumor antigens, thereby enabling the exploitation of discovery of autoantibodies as cancer biomarkers (36–40). Several autoantibodies against tumor antigens, such as p53, ubiquilin 1, cyclin Y, livin, and survivin, have been found to be readily detectable in serum samples collected from LC patients (41–45). However, previous reports of identification of LC biomarkers suffered from small sample sizes, a lack of a proper disease control group, and/or limited subtypes of LC (45–47). As a result, to date, these reported autoantibody-based serological biomarkers do not provide sufficient sensitivity or specificity for LC diagnosis, let alone early LC diagnosis (45, 48).

In summary, we performed a comprehensive autoantibody-based survey for the discovery and validation of serum biomarkers for early LC diagnosis. It is important to note that because the serum samples were collected from patients at diagnosis, the biomarkers identified in this study were not identified in a LC screening cohort. Therefore, it would be important in the future to examine the performance of these biomarkers with serum samples collected before a person shows any LC-relevant pulmonary symptoms. Furthermore, because some genes are known to be mutated in LC cancer, we believe that inclusion of mutated proteins on the protein arrays may further improve accuracy of LC diagnosis and reduce false positive rates. As compared with the protein-based biomarkers for cancer diagnosis, we believe that the HuProt array-based approach offers a unique advantage because the identified biomarkers are autoantibody-based. Because most proteins are not stable, especially when secreted into the peripheral, the concentrations of these proteins can fluctuate tremendously from individual to individual, making them unreliable to be detected. On the other hand, autoantibodies are extremely stable in the blood and can be amplified by the immune system. Indeed, autoantibodies of the IgG/A/E isotypes can have long lasting memories in a patient, rendering them ideal biomarkers for diagnosis and prognosis. Therefore, we believe that HuProt array-based approach is and will continue to play a dominant role in cancer biomarker identification.

## Supplementary Material

Supplemental Data

## Footnotes

^1^ The abbreviations used are:

LC: lung cancer
LBL: lung benign lesions
SCLC: small-cell lung cancer
NSCLC: nonsmall-cell lung cancer
CT: computed tomography
COPD: chronic obstructive pulmonary disease
TB: pulmonary tuberculosis
RC: rectal cancer
LiC: liver cancer
CC: cervical cancer
EC: esophagus cancer
GC: gastric cancer
p53: tumor protein p53
HRas: HRas proto-oncogene, GTPase
ETHE1: ETHE1 persulfide dioxygenase.
