# Supplementary material for: Identification of Serological Biomarkers for Early Diagnosis of Lung Cancer Using a Protein Array-Based Approach
Source: Mol Cell Proteomics. 2017 Oct 11;16(12):2069–78. doi: 10.1074/mcp.RA117.000212 (PMC5724172; doi:10.1074/mcp.RA117.000212)
Supplement: Supplemental Data [file supp_16_12_2069__index.html]

Identification of serological biomarkers for early diagnosis of lung cancer using a protein array-based approach — Novel lung cancer biomarkers by human proteome microarrays — Identification of Serological Biomarkers for Early Diagnosis of Lung Cancer Using a Protein Array-Based Approach — Novel Lung Cancer Biomarkers by Human Proteome Microarrays — Supplemental Data 

# Identification of Serological Biomarkers for Early Diagnosis of Lung Cancer Using a Protein Array-Based Approach

## Supplemental Data

- Supplementary Figure S1 - Eight biomarkers discovered and validated in Phase II.
- Supplementary Table S1 - Characteristics of all samples used in this study.
- Supplementary Table S2 - Candidates identified from HuProt Array.
- Supplementary Table S3 - Functional enrichment analysis of 170 candidate proteins
