# Supplementary figures and images for: Identification of Serological Biomarkers for Early Diagnosis of Lung Cancer Using a Protein Array-Based Approach
Source: Mol Cell Proteomics. 2017 Oct 11;16(12):2069–78. doi: 10.1074/mcp.RA117.000212 (PMC5724172; doi:10.1074/mcp.RA117.000212)

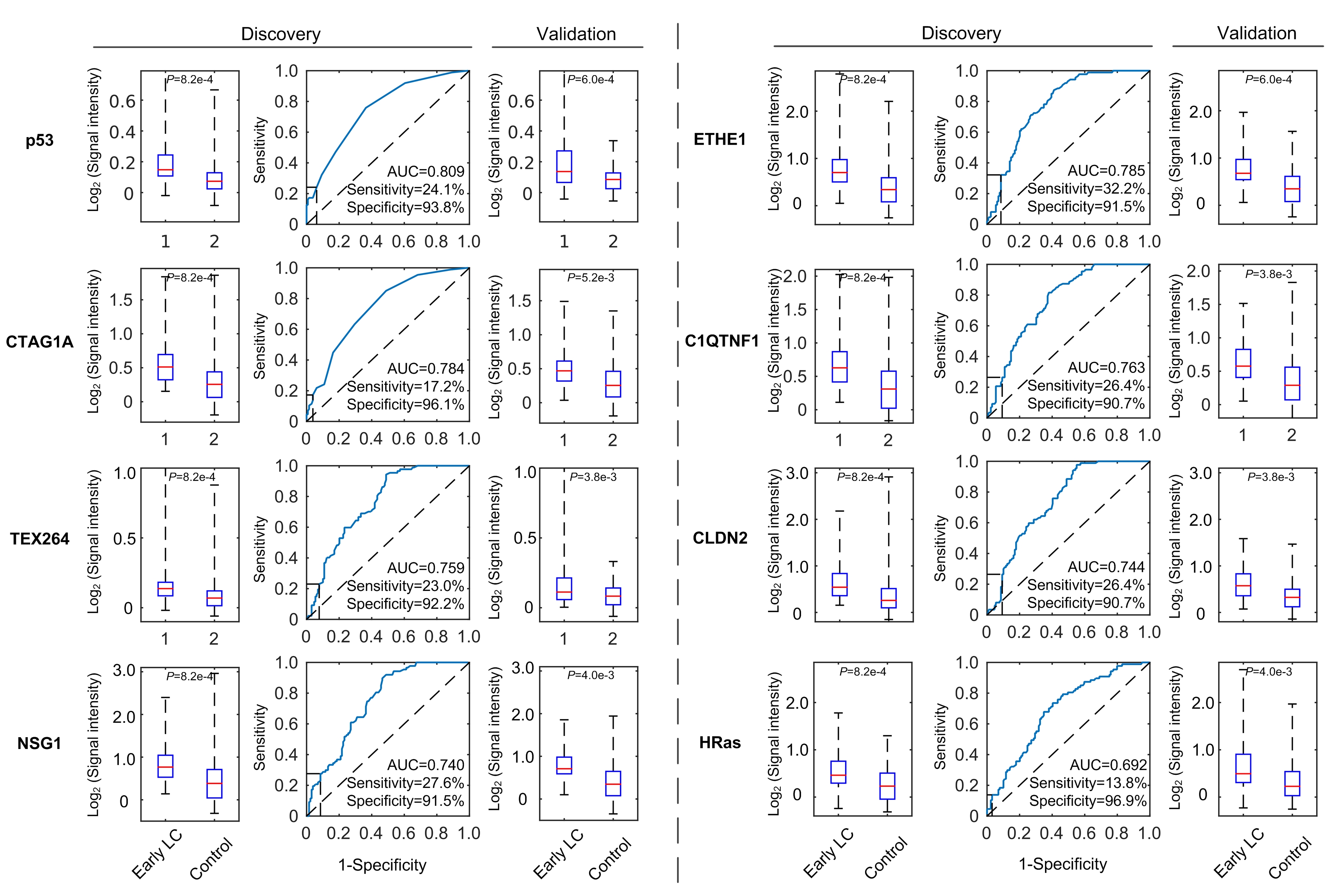

Supplement: Supplemental Data [file supp_RA117.000212_4815_1_supp_10487_rxcsyz.jpg]
